# Point-of-Care System for HTLV-1 Proviral Load Quantification by Digital Mediator Displacement LAMP

**DOI:** 10.3390/mi12020159

**Published:** 2021-02-05

**Authors:** Lisa Becherer, Jacob Friedrich Hess, Sieghard Frischmann, Mohammed Bakheit, Hans Nitschko, Silvina Stinco, Friedrich Zitz, Hannes Hofer, Giampiero Porro, Florian Hausladen, Karl Stock, Dominik Drossart, Holger Wurm, Hanna Kuhn, Dominik Huber, Tobias Hutzenlaub, Nils Paust, Mark Keller, Oliver Strohmeier, Simon Wadle, Nadine Borst, Roland Zengerle, Felix von Stetten

**Affiliations:** 1 Hahn-Schickard, Georges-Koehler-Allee 103, 79110 Freiburg, Germany; Lisa.Becherer@Hahn-Schickard.de (L.B.); Jacob.Hess@Hahn-Schickard.de (J.F.H.); hanna.k.kuhn@googlemail.com (H.K.); Dominik.Huber@imtek.uni-freiburg.de (D.H.); Tobias.Hutzenlaub@Hahn-Schickard.de (T.H.); Nils.Paust@Hahn-Schickard.de (N.P.); Mark.Keller@spindiag.de (M.K.); oliver.strohmeier@spindiag.de (O.S.); simon.wadle@volpi-group.com (S.W.); Nadine.Borst@Hahn-Schickard.de (N.B.); Roland.Zengerle@Hahn-Schickard.de (R.Z.); 2 Laboratory for MEMS Applications, IMTEK—Department of Microsystems Engineering, University of Freiburg, Georges-Koehler-Allee 103, 79110 Freiburg, Germany; 3 Mast Diagnostica GmbH, Feldstraße 20, 23858 Reinfeld, Germany; frischmann@mast-diagnostica.de (S.F.); bakheit@mast-diagnostica.de (M.B.); 4 Department of Virology, Pettenkoferstraße 9a, Max von Pettenkofer-Institute, 80336 Munich, Germany; nitschko@mvp.lmu.de (H.N.); stinco.silvina@gmail.com (S.S.); 5 E.L.T. Kunststofftechnik & Werkzeugbau GmbH, Weidenweg 339, 8240 Friedberg, Austria; f.zitz@elt.at (F.Z.); h.hofer@elt.at (H.H.); 6 Datamed srl, Via Achille Grandi 4/6, 20068 Peschiera Borromeo MI, Italy; giampiero.porro@datamedsrl.com; 7 Institut für Lasertechnologien in der Medizin und Meßtechnik, University of Ulm, Helmholtzstraße 12, 89081 Ulm, Germany; florian.hausladen@ilm-ulm.de (F.H.); karl.stock@ilm-ulm.de (K.S.); dominik.drossart@ilm-ulm.de (D.D.); holger.wurm@ilm-ulm.de (H.W.); 8 NB Technologies GmbH, Ludwig-Erhard-Allee 2, 53175 Bonn, Germany; 9 Spindiag GmbH, Engesserstraße 4a, 79108 Freiburg, Germany; 10 Volpi AG, Wiesenstrasse 33, 8952 Schlieren, Switzerland

**Keywords:** human T-cell lymphotropic virus 1, digital loop-mediated isothermal amplification, point-of-care testing, centrifugal microfluidics, proviral load measurements

## Abstract

This paper presents a universal point-of-care system for fully automated quantification of human T-cell lymphotropic virus type 1 (HTLV-1) proviral load, including genomic RNA, based on digital reverse RNA transcription and c-DNA amplification by MD LAMP (mediator displacement loop-mediated isothermal amplification). A disposable microfluidic LabDisk with pre-stored reagents performs automated nucleic acid extraction, reaction setup, emulsification, reverse transcription, digital DNA amplification, and quantitative fluorogenic endpoint detection with universal reporter molecules. Automated nucleic acid extraction from a suspension of HTLV-1-infected CD4+ T-lymphocytes (MT-2 cells) yielded 8 ± 7 viral nucleic acid copies per MT-2 cell, very similar to the manual reference extraction (7 ± 2 nucleic acid copies). Fully automated sample processing from whole blood spiked with MT-2 cells showed a comparable result of 7 ± 3 copies per MT-2 cell after a run time of two hours and 10 min.

## 1. Introduction

The human T-cell lymphotropic virus type 1 (HTLV-1) is a highly carcinogenic oncovirus affecting an estimated 5–20 million people worldwide [1]. HTLV-1 infections can be found throughout the world, with high prevalence in the southwestern part of Japan, sub-Saharan Africa, Latin America, and the Caribbean [2]. HTLV-1 is endemic primarily in resource-constrained settings lacking infrastructure [3,4]. Hence, there is a great need for a point-of-care testing (POCT) system that facilitates access to HTLV-1 nucleic acid testing. The virus is associated with an aggressive CD4^+^ adult T-cell lymphoma/leukaemia (ATLL) and inflammatory diseases such as HTLV I uveitis (HU) [5], infective dermatitis [6], arthropathy [7], and HTLV-1-associated myelopathy/tropical spastic paraparesis (HAM/TSP). Correlations between proviral load and the outbreak of HU [8] and HAM/TSP [9,10] have already been discovered. Further studies have also shown that the HTLV-1 *tax* messenger RNA (mRNA) level, which reflects viral expression, correlates with disease progression and severity in HAM/TSP patients [11]. Consequently, monitoring of proviral loads may play an essential role in controlling the pathogenesis of HTLV-1-associated diseases. Current lab-based techniques for HTLV-1 proviral load measurement rely on quantitative polymerase chain reaction (PCR) [12,13], which is an unideal candidate for use in a point-of-care setting. PCR requires precise thermocycling, which is time-consuming and necessitates sophisticated instrumentation. For POCT, isothermal approaches, e.g., loop-mediated isothermal amplification (LAMP), provide faster time to result options and enable robust nucleic acid testing with moderate technological effort [14]. It has been shown that isothermal protocols in combination with digital amplification can provide precise quantification, circumventing the need for standard curves [15].

A significant challenge in the development of a POCT test for HTLV-1 is the integration of sample preparation from whole blood, due to the virus’s low abundance in the plasma of infected individuals [16]. This is because HTLV-1 is strongly cell-associated, and often only a small fraction of lymphocytes are found to be infected. Centrifugal microfluidics has proved advantageous when shrinking entire laboratory workflows to the size of a small diagnostic cartridge, enabling fully automated sample-to-answer tests at the point of care [17]. In this work, we present a novel centrifugal POCT test for the automated, nucleic acid-based quantification of HTLV-1 directly from whole blood. Originally, this digital POC sample-to-answer platform was introduced for the rapid detection of species and resistance markers in methicillin-resistant Staphylococcus aureus (MRSA) on a single-cell level [18]. By adding additional sample pre-processing workflow steps, such as a bead-based extraction module, even more challenging laboratory protocols can be automated on the same digital POC sample-to-answer platform. The centrifugal microfluidic *LabDisk* cartridge for HTLV-1 testing integrates the complete workflow: insertion of a finger prick blood sample into the *LabDisk*, nucleic acid extraction with subsequent digital reverse transcription (RT-)LAMP (dLAMP), and fluorescence readout. The *LabDisk* cartridge enables automated liquid handling and contains pre-stored reagents for nucleic acid extraction, reverse transcription of RNA, and LAMP. This integrated functionality in combination with the benefits of absolute quantification by digital mediator displacement (MD) LAMP demonstrate the potential for the next innovation cycle of POC analysis and could, with appropriate development, replace state-of-the-art instruments such as the Alere™ q (Abbott Laboratories, Chicago, IL, USA) for HIV-1/2, the SAMBA^®^ (Diagnostics for the real world Ltd., San Jose, CA, USA), the Xpert VL (Cepheid, Sunnyvale, CA, USA) for HIV-1 and hepatitis B/C, or the EOSCAPE-HIV (Wave80 Biosciences, San Francisco, CA, USA) for HIV-1.

## 2. Materials and Methods

### 2.1. Cell Cultures, Viral Strains, and Sample Material

Genomic HTLV-1 nucleic acids were isolated from cell cultures (HTLV-1-infected CD4^+^ T-lymphocytes, designated as MT-2 cells [19]) with MagaZorb DNA Mini-Prep Kit (Promega, Fitchburg, WI, USA). The concentration of MT-2 cells was analysed by cell counting. For sample-to-answer testing, 3150 and 9600 MT-2 cells from culture were spiked to 50 µL of fresh whole blood to simulate proviral loads of 63,000 and 192,000 copies/mL blood, respectively.

### 2.2. dLAMP: Oligonucleotides and LAMP Reaction

The dLAMP assay includes the reverse transcription of viral RNA, the amplification of c-DNA and proviral DNA, and the sequence-specific detection of LAMP amplicons by mediator displacement (MD). The primers and the fluorescent universal reporter for the dLAMP are taken from [20] and listed in the Electronic Supplementary Materials (ESI) in Table S1. A 50 µL reaction mix prepared with fresh reagents contained 25 µL 1× RM MPM buffer (Mast Diagnostica GmbH, Reinfeld, Germany), 16 U *Bst* 2.0 WarmStart DNA Polymerase (New England Biolabs, Frankfurt, Germany), 20 U Transcriptor Reverse Transcriptase (Roche Diagnostics, Mannheim, Germany), primers (1.6 μM each of FIP and BIP, 0.6 μM LF, 0.2 μM LF_Medc, and 0.2 μM each of F3 and B3), 0.1 μM mediator, and 0.05 μM universal reporter (biomers.net, Ulm, Germany). For the dLAMP, the reaction mix was incubated for one hour at 64 °C.

### 2.3. Functional Model of the POCT Instrument

**Figure 1:**
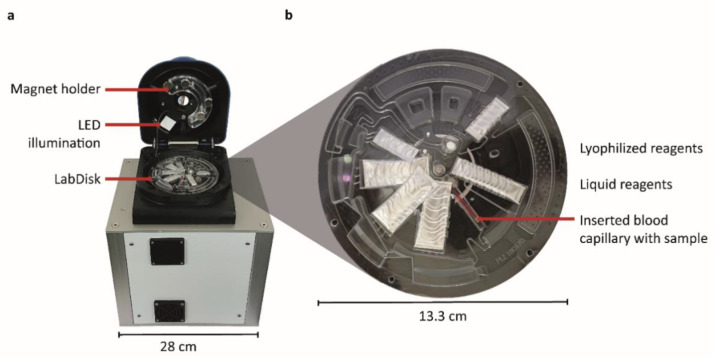
Photographs of (**a**) the standalone point-of-care testing (POCT) instrument with centrifuge, incubator and fluorescence camera and (**b**) the microthermoformed microfluidic LabDisk cartridge. A detailed overview of the LabDisk cartridge and its pre-stored reagents is depicted in Figure 2.

Microfluidic actuation, incubation, and fluorescence readout were conducted in a functional model of a standalone POCT instrument (Figure 1). Rotation, heating, and positioning for readout were performed in a LabDisk-Player 1 instrument (QIAGEN Lake Constance GmbH, Stockach, Germany), which was augmented with an optical unit for the fluorescence detection of droplets (Figures S1 and S2). This unit provides sequentially switched light-emitting diodes (LEDs) (Table S4) and filters for excitation, as well as selected emission filters (Table S5). For the excitation, oligonucleotides specific for HTLV-1 were labelled with the green fluorescent dye, FAM, and excited with an LED at 465 nm. Further technical information is provided in [18].

### 2.4. LabDisk Cartridge Manufacturing Including Reagent Pre-Storage

**Figure 2:**
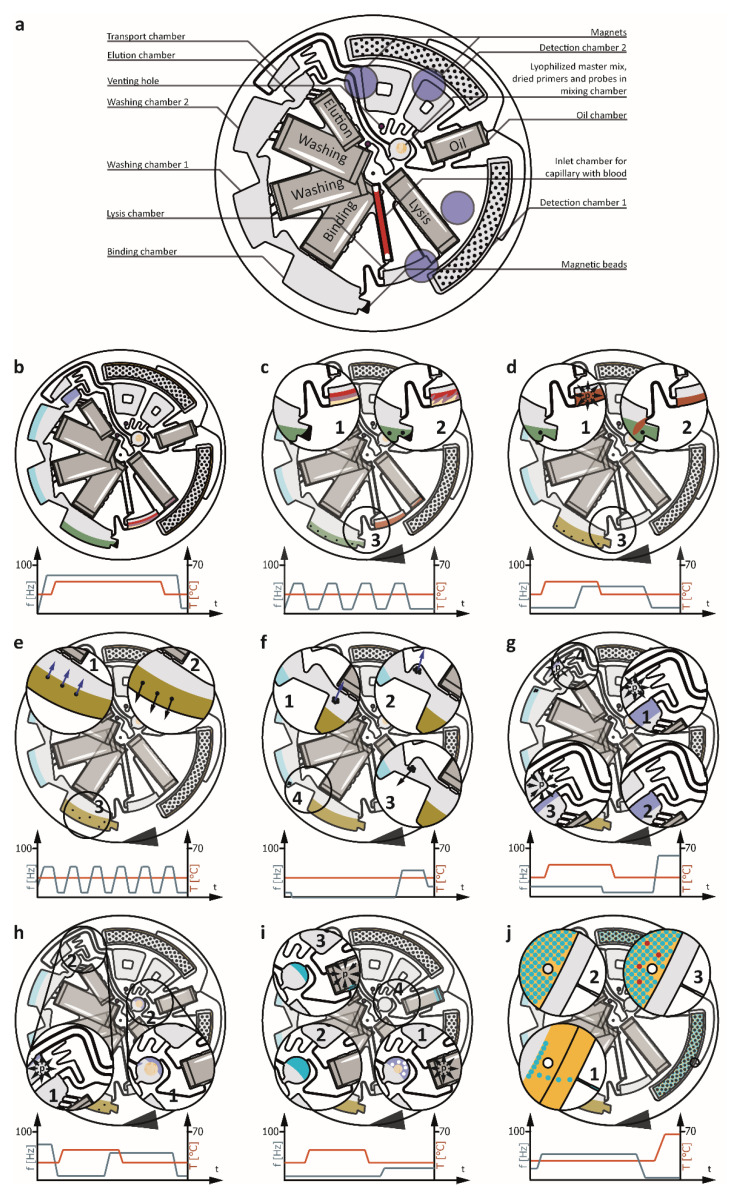
Complete workflow of the LabDisk. Blue arrows depict magnetic forces, whereas centrifugal forces are depicted by black arrows. Pneumatic pressure is represented by the letter p encircled with arrows. (**a**) Overview of the cartridge and pre-stored reagents. (**b**) Liquid release from stick-packs. (**c**) Lysis. (**d**) Liquid transport into the binding chamber. (**e**) Binding. (**f**) Magnetic bead transfer. (**g**,**h**) Elution transport into mixing chamber. (**i**) Pre-stored reagent mixing and transport into oil chamber. (**j**) Droplet generation by step emulsification and subsequent dLAMP.

The microfluidic *LabDisk* cartridges were manufactured by soft lithography in the Hahn–Schickard prototyping facilities [21]. In brief, the master tool for replication was manufactured out of Plexiglas poly(methyl methacrylate) (PMMA) (Evonik, Darmstadt, Germany) on a KERN Evo mill (KERN Microtechnik, Eschenlohe, Germany). A negative tool was produced by casting Elastosil RT-607 polydimethylsiloxane (PDMS) (Wacker Chemie, Haigerloch, Germany). Replication of the *LabDisk* cartridges was achieved by microthermoforming 300 µm COC 6013/8007 foils (TOPAS Advanced Polymers, Frankfurt, Germany) using a blow moulding process, which was implemented with a HEX01 hot embossing machine (Jenoptik, Villingen-Schwenningen, Germany). After microthermoforming, all pre-stored reagents were deposited into the *LabDisk* cartridge. Pressure-sensitive adhesive foil 9795R (3M, Neuss, Germany) was used to seal the microfluidic cartridge. All the reagents, except the proteinase K, are pre-stored on the microfluidic cartridge as depicted in Figure 2a. So-called stick-packs [22] contain the liquid reagents for the DNA purification, namely, the lysis, binding, washing, and elution buffers, as well as the oil for the droplet emulsification. The master mix for the dLAMP is stored in lyophilized form next to the dried Tris, primers, and universal reporter in the mixing chamber. Dried magnetic beads (MagaZorb DNA Mini-Prep Kit) are pre-stored in the binding chamber. Details on the cartridge assembly, including reagent pre-storage, are presented in the ESI in Table S2.

### 2.5. LabDisk Cartridge Automation Principle

The sample is taken directly from the finger of a patient using a plastic capillary coated with ethylenediaminetetraacetic acid (EDTA) (Sanguis Counting GmbH, Nümbrecht, Germany) that can collect up to 50 µL of whole blood. Thanks to the pre-storage concept, the user only has to insert the blood-filled capillary into the *LabDisk* (Figure 2a) and inject 20 µL of proteinase K solution (MagaZorb DNA Mini-Prep Kit). Afterwards, the inlet hole is sealed with pressure-sensitive adhesive tape (9795R), the *LabDisk* is inserted into the POCT instrument, and the fully automated workflow (see ESI Table S3) starts.

First, all the stick-packs are opened by the increases in frequency of rotation and temperature. The elevated pressure inside the stick-packs peels them open and releases the reagents [22]. At the same time, the whole blood sample and the proteinase K are transported by centrifugal force into the lysis chamber (Figure 2b). When all the stick-packs have released their reagents, the temperature is decreased to room temperature. The subsequent lysis is performed with continuous shake-mode mixing (Figure 2c, [23]) to allow even distribution of all the reagents within the lysis chamber. Simultaneously, the pre-stored magnetic beads come into contact with the binding buffer and start to resuspend. After lysis, the temperature is increased while keeping the rotational frequency low to generate pneumatic pressure inside the lysis chamber and prime the siphon connecting it to the binding chamber [24] (Figure 2d). As soon as all the liquid has left the channel connecting the capillary inlet to the binding chamber, the lysis chamber is vented and all the remaining liquid is transported into the binding chamber [25]. During binding, the rotational frequency is periodically reduced and increased. At low frequencies, magnetic forces outmatch centrifugal forces and pull all magnetic particles in a radially inward direction. At high frequencies, the magnetic beads are centrifuged towards the outer edge of the binding chamber. This interplay of forces allows efficient mixing during binding (Figure 2e). Next, the magnetic beads are transported with the help of magnets into washing chamber 1. This is done by moving the cartridge stepwise underneath static magnets positioned above the *LabDisk* (Figure 2f) [26]. As soon as the beads have collected at the radially inward part of the binding chamber, the rotational frequency is increased and all the captured beads are centrifuged into washing chamber 1. An example of the bead transfer steps is presented in the ESI in Figure S3. The magnet-induced mixing, as well as the described magnetic particle handling (Figure 2e,f), are repeated for the elution and both washing steps. After elution, the magnetic particles are transported back into washing chamber 2 using the same magnetic particle handling principle to avoid unwanted bead transfer into the mixing chamber. At this point, all the extracted DNA is inside the elution buffer and needs to be transported into the mixing chamber. This is done by increasing the temperature (Figure 2g), which leads to an overpressure in the transport chamber. This pressure is slowly reduced via a venting hole [24] with a high fluidic resistance. When equilibrium is reached, the temperature is decreased. This reduces the volume of the air inside the cartridge and, thereby, generates an underpressure inside the transport chamber. As the venting hole and the transport chamber are only connected by a channel with high fluidic resistance, the liquid is sucked over the siphon connecting the elution and transport chambers. The rotational frequency is then increased, transferring all the liquid into the transport chamber. Liquid entering the transport chamber compresses the air and generates pneumatic pressure [27] (Figure 2h). Simultaneously reducing the rotational frequency and increasing the temperature transports the liquid into the mixing chamber, as the high fluidic resistance of the channel connecting the elution and transport chambers prevents liquid transport back into the elution chamber [28]. The liquid entering the mixing chamber immediately starts to dissolve the pre-stored dLAMP reagents. However, to guarantee homogenous mixing and to avoid reagent concentration peaks inside the liquid, a bubble mixing step is performed [29]. To achieve this, the temperature is increased and the pressure builds in the detection and oil chambers, as well as in the auxiliary air volume next to the oil chamber. The high fluidic resistance of the venting channel slows down the equalization of the pressure to the ambient air pressure, and the overpressure transports pressurized air through the connection channel into the mixing channel. At this point, bubbles form, ascend due to buoyancy, and thereby mix the fluid (Figure 2i). Subsequently, the temperature is reduced, the siphon between the mixing and the oil chambers is primed [24], and the liquid is transferred into the oil chamber. An elevated rotational frequency allows the liquid to be transported into the detection chambers. Centrifugal step emulsification [30] generates droplets with a diameter of 100 µm (Figure 2j). A stroboscopic image of the droplet generation is depicted in the ESI in Figure S4. A monolayer array of droplets is generated from a volume of 50 µL (elution buffer). A stroboscopic photo of the monolayer and the droplets is presented in Figure S5. The dispersion and diameter of the droplets is estimated based on microscopic and stroboscopic photos. However, some liquid (~0.5 µL) remains in the channels connecting the detection chamber with the preceding microfluidic structures. The reduced total volume in combination with the droplet diameter results in approximately 95,000 droplets. The dLAMP is performed at a low rotational frequency.

## 3. Results and Discussions

### 3.1. Quantification of Nucleic Acids per MT-2 Cell with Fresh and Dried Reagents

**Figure 3:**
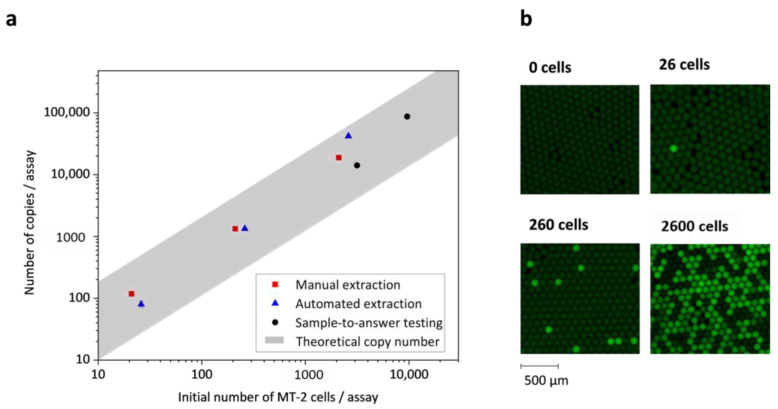
(**a**) The number of copies per assay determined (1) by digital loop-mediated isothermal amplification (dLAMP) of human T-cell lymphotropic virus type 1 (HTLV-1) with fresh reagents and manually extracted nucleic acids (red squares), (2) by dLAMP of HTLV-1 with fresh reagents and automated extraction of nucleic acids (blue triangles), and (3) by fully automated sample-to-answer testing (black circles). The calculated number of copies per assay is plotted against the theoretical CD4+ T-lymphocytes (MT-2) cell number per assay. The grey area illustrates the interval of nucleic acid copies per MT-2 cell given in the literature and ranges from 1 to 16 copies per MT-2 cell. (**b**) Crops of fluorescence images from the dLAMP of HTLV-1 for different MT-2 cell numbers per assay.

A reliable estimation of the average copy number of HTLV-1 nucleic acids per MT-2 cell appears difficult, because the process of HTLV-1 replication in vivo remains poorly understood. Reports in the literature are inconsistent, and values vary between a single proviral copy per MT-2 cell [31,32] and more than 10, 12 [33], or 16 [34] copies per MT-2 cell. According to the variance given in the literature, the theoretical number of nucleic acid copies per MT-2 cell is highlighted as a grey area ranging from 1 to 16 copies per MT-2 cell (Figure 3). To determine the number of HTLV-1 copies per MT-2 cell, we conducted a dLAMP of HTLV-1 with fresh reagents on a microfluidic chip (Figure S6) as described previously [30]. Zero, 21, 210, and 2100 MT-2 cells per assay were extracted manually with MagaZorb DNA Mini-Prep Kit. The eluate was added to the dLAMP mix, the solution was emulsified by centrifugal step emulsification, and it was then incubated at dLAMP conditions. Positive droplets were counted after incubation and the number of nucleic acid molecules was calculated by Poisson statistics. The average number of HTLV-1 copies per MT-2 cell was 7 ± 2, which is within the range given by the literature. This experiment served as a reference method for the assessment of (1) the performance of dried reagents, (2) the efficiency of extraction on the *LabDisk*, and (3) the performance of fully automated sample-to-answer testing. The dLAMP assay performance with dried reagents, including pellets of lyophilized reagents, primers, MD oligonucleotides, and Tris, was investigated with manually extracted HTLV-1 nucleic acids using the microfluidic chip, and yielded results (7 ± 5 copies per MT-2 cell) comparable to the reference method with fresh reagents. The total number of copies per assay is plotted against the initial MT-2 cell number per assay in Figure 3.

### 3.2. Efficiency of Automated Nucleic Acid Extraction and Purification on LabDisk

The efficiency of automated extraction on the *LabDisk* was assessed by mixing 5 µL of MT-2 cells from fresh culture with 45 µL of whole blood to reach a total volume of 50 µL. The mix was collected in a capillary and transferred into the sample inlet chamber on the *LabDisk* (Figure 2). Automated extraction was conducted according to the described protocol (ESI Table S3), but stopped after elution. Here, 5 µL of the 50 µL of eluate collected after extraction was tested separately with fresh reagents by dLAMP on a microfluidic chip. The performance of the extraction on the *LabDisk* was thus assessed without the influence of pre-stored reagents. Automated extraction was tested with 0, 26, 260, and 2600 MT-2 cells per assay, and yielded an average of 8 ± 7 copies per MT-2 cell. The large error is ascribed to a high outlier, namely the 2600 MT-2 cell data point. The efficiency of automated nucleic acid extraction on the *LabDisk* is comparable to the manual reference extraction. The total number of copies per assay is plotted against the initial MT-2 cell number per assay in Figure 3.

### 3.3. Sample-to-Answer Testing with HTLV-1-Infected Lymphocytes

After verifying the functionality of rehydrated reagents and the efficiency of extraction on the *LabDisk*, fully automated sample-to-answer testing was performed with whole blood (45 µL) spiked with MT-2 cells from fresh culture (5 µL). According to reports in the literature [35], the number of HTLV-1-infected lymphocytes (per 50 µL blood) varies between HAM/TSP patients (11,630 infected lymphocytes), HU patients (3840 infected lymphocytes), and asymptomatic carriers (540 infected lymphocytes) (calculation in the ESI). Therefore, we tested 3150 and 9600 MT-2 cells per assay to reflect the number of infected lymphocytes in individuals with an HTLV-1 infection. The automated sample-to-answer testing yielded 7 ± 3 copies per MT-2 cell, and thus confirmed the high efficiency of the microfluidic workflow.

The POCT test presented herein detects the provirus (DNA) integrated into the genome of the human host cell as well as viral RNA. As HTLV-1 exists mainly as provirus, there is no detectable virus in the blood plasma or the host cells, and thus the detected RNA indicates the HTLV-1 *tax* mRNA level, which correlates with disease progression. In HAM/TSP patients, an increased HTLV-1 *tax* mRNA level is accompanied by an increased HTLV-1 proviral load, which is also a marker for disease outbreak [11]. However, differences were found for ATLL patients showing low HTLV-1 *tax* mRNA levels, but high proviral loads. Consequently, in the latter case, it would be advantageous to differentiate between the mRNA level and the proviral load, for example, through selective amplification of DNA by omitting reverse transcription or by selective amplification of RNA [36,37].

The processing time for the automated sample-to-answer testing was 2 h 10 min, including 60 min for nucleic acid extraction, 60 min for dLAMP, and 10 min for readout of the fluorescence signals. The presented system requires at least twice as long as currently available systems, such as the Alere™ q for the detection of HIV-1/2, which requires approximately 55 min processing time. The potential to reduce the processing time lies in the adjustment of the dLAMP reagent composition and the extraction steps.

## 4. Conclusions

In the present work, we demonstrated a POCT system for fully automated, digital HTLV-1 proviral load quantification from whole blood based on a centrifugal microfluidic *LabDisk* platform. The automation and miniaturization of the laboratory workflow, including nucleic acid extraction, reverse transcription, and digital amplification with integrated fluorescence readout, reduces manual handling steps to the insertion of the blood capillary and the proteinase solution, thereby providing a quantitative HTLV-1 test for the point of care. Precise quantification is achieved by digital mediator displacement reverse transcription LAMP (MD RT-LAMP), removing any need for standard curves. The quantified HTLV-1 proviral load in blood samples spiked with infected MT-2 cells was comparable to a manual reference test (7 ± 3 vs. 7 ± 2 nucleic acid copies per MT-2 cell). The results were available after two hours and 10 min. Our novel system stands out because of its ability to provide calibration-free, digital sample-to-answer quantification at the point of care, as well as for its potential to be adapted for the implementation of different microfluidic applications.

Further steps towards the development of a commercial product should include the pre-storage of proteinase K in the inlet chamber or in the blood capillary in dried form. In addition, the time to result should be reduced to <60 min. This can be achieved by optimizing the dLAMP, microfluidic workflow, or POCT instrument. A significant reduction of the incubation time of the dLAMP to less than 25 min can be achieved by adjusting the enzyme concentration, titrating primer concentrations, or using enhancing LAMP additives. Another approach deals with the time reduction of single steps in the microfluidic workflow. For instance, all bead handling holding times were kept as defined by the manufacturer in the manual reference workflow. However, efficient mixing within the microfluidic structure could decrease the binding, washing, and elution times and result in a lower total time. Heating steps by the POCT instrument were quite time-consuming and an improvement of the heating system could decrease the total workflow time. However, these steps were not part of this work. Furthermore, the expansion of the assay to additional targets could allow the detection of co-infections, e.g., HTLV-1/HIV.

## Supplementary Materials

The following are available online at https://www.mdpi.com/2072-666X/12/2/159/s1, Table S1: Primer and MD oligo sequences; Table S2: Cartridge manufacturing steps including all pre-stored reagents; Table S3: Complete parameter protocol for microfluidic cartridge; Figure S1: Schematic depiction of the optical path; Figure S2: 3D-CAD inside view of the POCT instrument; Table S4: Specifications of the LEDs; Table S5: Bandpass filters used to filter LED light; Figure S3: Stroboscopic images of the bead transfer; Figure S4: Stroboscopic image of the droplet generation; Figure S5: Stroboscopic image of the generated droplets; Figure S6: Layout of the microfluidic chip.
