# Supplementary material for: Point-of-Care System for HTLV-1 Proviral Load Quantification by Digital Mediator Displacement LAMP
Source: Micromachines (Basel). 2021 Feb 5;12(2):159. doi: 10.3390/mi12020159 (PMC7915047; doi:10.3390/mi12020159)
Supplement: Supplementary file 1 [file micromachines-12-00159-s001.pdf]

micromachines

Supplementary Materials: Point-of-Care System for HTLV-1 Proviral Load Quantification by Digital Mediator Displacement LAMP

Lisa Becherer [1,2,†], Jacob Friedrich Hess [1,2,†], Sieghard Frischmann [3], Mohammed Bakheit [3], Hans Nitschko [4], Silvina Stinco [4], Friedrich Zitz [5], Hannes Hofer [5], Giampiero Porro [6], Florian Hausladen [7], Karl Stock [7], Dominik Drossart [7], Holger Wurm [7], Hanna Kuhn [1,8], Dominik Huber [1,2], Tobias Hutzenlaub [1,2], Nils Paust [1,2], Mark Keller [1,2,9], Oliver Strohmeier [1,2,9], Simon Wadle [1,2,10], Nadine Borst [1,2], Roland Zengerle [1,2], and Felix von Stetten [1,2,*]

1 Hahn-Schickard, Georges-Koehler-Allee 103, 79110 Freiburg, Germany; Lisa.Becherer@Hahn-Schickard.de (L.B.); Jacob.Hess@Hahn-Schickard.de (J.F.H.); hanna.k.kuhn@googlemail.com (H.K.); Dominik.Huber@imtek.uni-freiburg.de (D.H.); Tobias.Hutzenlaub@Hahn-Schickard.de (T.H.); Nils.Paust@Hahn-Schickard.de (N.P.); Mark.Keller@spindiag.de (M.K.); oliver.strohmeier@spindiag.de (O.S.); simon.wadle@volpi-group.com (S.W.); Nadine.Borst@Hahn-Schickard.de (N.B.); Roland.Zengerle@Hahn-Schickard.de (R.Z.)
2 Laboratory for MEMS Applications, IMTEK—Department of Microsystems Engineering, University of Freiburg, Georges-Koehler-Allee 103, 79110 Freiburg, Germany
3 Mast Diagnostica GmbH, Feldstraße 20, 23858 Reinfeld, Germany; frischmann@mast-diagnostica.de (S.F.); bakheit@mast-diagnostica.de (M.B.)
4 Department of Virology, Pettenkoferstraße 9a, Max von Pettenkofer-Institute, 80336 Munich, Germany; nitschko@mvp.lmu.de (H.N.); stinco.silvina@gmail.com (S.S.)
5 E.L.T. Kunststofftechnik & Werkzeugbau GmbH, Weidenweg 339, 8240 Friedberg, Austria; f.zitz@elt.at (F.Z.), h.hofer@elt.at (H.H.)
6 Datamed srl, Via Achille Grandi 4/6, 20068 Peschiera Borromeo MI, Italy; giampiero.porro@datamedsrl.com
7 Institut für Lasertechnologien in der Medizin und Meßtechnik, University of Ulm, Helmholtzstraße 12, 89081 Ulm, Germany; florian.hausladen@ilm-ulm.de (F.H.); karl.stock@ilm-ulm.de (K.S.); dominik.drossart@ilm-ulm.de (D.D.); holger.wurm@ilm-ulm.de (H.W.)
8 NB Technologies GmbH, Ludwig-Erhard-Allee 2, 53175 Bonn, Germany
9 Spindiag GmbH, Engesserstraße 4a, 79108 Freiburg, Germany
10 Volpi AG, Wiesenstrasse 33, 8952 Schlieren, Switzerland
* Correspondence: Felix.von.Stetten@Hahn-Schickard.de; Tel.: +49-761-203-73243
† Authors contributed equally.

Citation: Becherer, L.; Hess, J.F.; Frischmann, S.; Bakheit, M.; Nitschko, H.; Stinco, S.; Zitz, F.; Hofer, H.; Porro, G.; Hausladen, F.; et al. Point-of-Care System for HTLV-1 Proviral Load Quantification by Digital Mediator Displacement LAMP. *Micromachines* **2021**, *12*, 159. https://doi.org/10.3390/mi12020159

Received: 18 January 2021
Accepted: 2 February 2021
Published: 5 February 2021

Publisher's Note: MDPI stays neutral with regard to jurisdictional claims in published maps and institutional affiliations.

Copyright: © 2021 by the authors. Licensee MDPI, Basel, Switzerland. This article is an open access article distributed under the terms and conditions of the Creative Commons Attribution (CC BY) license (http://creativecommons.org/licenses/by/4.0/).

Table S1. Primer and MD oligo sequences. The concentrations are given for dLAMP with pre-stored oligonucleotides.

Target	Description	Sequence (5′–3′) [1]	Concentration per 50 µl assay
HTLV-1 (*tax* gene)	HTLV-F3	CCATCGATGGACGCGTTAT	0.25 µM
	HTLV-B3	TATTTGCGCATGGCCTGG	0.25 µM
	HTLV-FIP	AGAGGTTCTCTGGGTGGGGACGGCTCAGCTCTACAG-TTC	2.00 µM
	HTLV-BIP	AGACCCTCAAGGTCCTTACCCCGAAGGAGGGTG-GAATGTTGG	2.00 µM
	HTLV-LF	GAGGGGAGTCGAGGGATAAG	0.75 µM
	HTLV-LB	-	
	HTLV_LF _Medc	GGTCGTAGAGCCCATTGCGCGATGAGTGGGAGGGGAG-TCGAGGGATAAG	0.25 µM
	Mediator	CCACTCATCGCGCAATGGGCTCTACGACC	0.13 µM
	Universal reporter	BMN-Q-535-ATTGCGGGAGATGAGACCCGCAA-dT-FAM-TGTTGGTCGTAGAGCCCAGAACGA-C3	0.2 µM

The concentrations of oligonucleotides for dLAMP with pre-stored reagents are 1.25-fold increased compared to dLAMP with fresh reagents.

Table S2. Cartridge manufacturing steps including all pre-stored reagents.

Step number	Step description
1	Thermoform upper cartridge part including all chambers and channels required for the microfluidic automation
2	Insert and dry oligonucleotides and Tris buffer in mixing chamber<br>Primer mix is prepared according to the concentrations listed in Table S1 (total volume of primer mix per *LabDisk* and dLAMP: 3.5 µl).<br>3.5 µl primer mix and 1 µl of 0.5 mM Tris are dried by Eppendorf Vacufuge (Eppendorf, Germany) for 15 min inside the mixing chamber of the *LabDisk*<br>Final concentration of Tris per assay (50 µl): 10 mM
3	Coat structures required for bead transfer with Teflon<br>Mix Teflon solution by combining Teflon with FC770 at a ratio of 1:199 (v:v)<br>Distribute 250 µl of the solution along the bead transfer structures<br>Wait for 5 minutes until Teflon solution dried
4	Insert dried beads at designated position in binding chamber<br>Mix PEG8000 solution by combining PEG8000 and water with a ratio of 1:1 (v:v)<br>Magazorb reagent (20 µl per *LabDisk*) is mixed with PEG8000 solution (10 µl per *LabDisk*)<br>The Magazorb/PEG8000 solution is dried in an oven for 55 min at 50 °C<br>The dried bead-pellet is positioned in the binding chamber
5	Insert stick-packs into the designated chambers<br>1x stick-pack with 200 µl lysis buffer (MagaZorb DNA Mini-Prep Kit, Promega, USA)<br>1x stick-pack with 500 µl binding buffer (MagaZorb DNA Mini-Prep Kit, Promega, USA)<br>2x stick-pack with 500 µl wash buffer each (MagaZorb DNA Mini-Prep Kit, Promega, USA)<br>1x stick-pack with 50 µl elution buffer (MagaZorb DNA Mini-Prep Kit, Promega, USA)<br>1x stick-pack with 50 µl fluorinated oil (HFE, Novec 7500 3 M Corp., USA with the addition of an interface stabilization agent Pico-Surf 1 5 %, Dolomite Ltd., United Kingdom)
6	Insert air filters into the two designated venting holes
7	Insert pellet into the mixing chamber
8	Seal cartridge twice with pressure sensitive adhesive sealing foil

Table S3. Complete parameter protocol for microfluidic cartridge.

Step number	Step description	Duration	Frequency	Acceleration deceleration	Temperature
1	Mix blood with proteinase	30 s	-30 Hz	5 Hz/s	Room temperature (RT)
2	Accelerate to peal stick-packs open	0 s	-55 Hz	5 Hz/s	"
3	Heat up to peal stick-packs open	60 s	"	-	55 °C
4	Cool down to room temperature	0 s	"	-	RT
5	Hold frequency until pressure is at atmospheric pressure	90 s	-40 Hz	5 Hz/s	"
6	Shake mode mixing of lysis reagents	1 s	-20 Hz	5 Hz/s	"
7	"	5 s	-15 Hz	5 Hz/s	"
8	Repeat steps 6 to 7 53 times	-	-	-	"
9	Decelerate for siphon priming	0 s	-5 Hz	15 Hz/s	"
10	Heat up for siphon priming	10 s	"	-	55 °C
11	Transfer liquid into binding chamber	15 s	-20 Hz	5 Hz/s	"
12	Accelerate until pressure is at atmospheric pressure	0 s	-40 Hz	5 Hz/s	"
13	Slowly cool down to avoid early siphon priming after elution chamber	60 s	"	-	45 °C
14	"	120 s	"	"	30 °C
15	Pull beads radially inwards	21 s	-8 Hz	5 Hz/s	"
16	Sediment beads	5 s	-15 Hz	5 Hz/s	"
17	Repeat steps 15 to 16 13 times	-	-	-	"
18	Initiate bead transfer	0 s	-5 Hz	5 Hz/s	"
19	Move cartridge until radial outward magnet is between lysis and binding chamber	15 s	-	-	"
20	Move cartridge underneath radial outward magnet with -0.5°/s until magnet is in between binding chamber and washing chamber 1	-	-	-	"
21	Hold position	10 s	0 Hz	-	"
22	Move cartridge until the same position is underneath a radial inward positioned magnet and hold	10 s	-	-	"
23	Move cartridge underneath radial inward magnet with -0.5°/s until magnet is at the centre of washing chamber 1	-	-	-	"
24	Transfer beads into washing chamber 1	0 s	-30 Hz	10 Hz/s	"
25	Pull beads radially inwards	8 s	-5 Hz	5 Hz/s	"
26	Sediment beads	3 s	-15 Hz	5 Hz/s	"
27	Repeat steps 25 to 26 39 times	-	-	-	"
28	Initiate bead transfer	0 s	-5 Hz	5 Hz/s	"
29	Move cartridge until radial outward magnet is between binding and washing chamber 1	15 s	-	-	"

30	Move cartridge underneath radial outward magnet with -0.5°/s until magnet is in between washing chamber 1 and 2	-	-	-	“
31	Hold position	10 s	0 Hz	-	“
32	Move cartridge until the same position is underneath a radial inward positioned magnet and hold	10 s	-	-	“
33	Move cartridge underneath radial inward magnet with -0.5°/s until magnet is at the centre of washing chamber 2	-	-	-	“
34	Transfer beads into washing chamber 2	0 s	-30 Hz	10 Hz/s	“
35	Pull beads radially inwards	8 s	-5 Hz	5 Hz/s	“
36	Sediment beads	3 s	-15 Hz	5 Hz/s	“
37	Repeat steps 35 to 36 39 times	-	-	-	“
38	Initiate bead transfer	0 s	-5 Hz	5 Hz/s	“
39	Move cartridge until radial outward magnet is between washing chamber 1 and 2	15 s	-	-	“
40	Move cartridge underneath radial outward magnet with -0.5°/s until magnet is in between washing chamber 2 and elution chamber	-	-	-	“
41	Hold position	10 s	0 Hz	-	“
42	Move cartridge until the same position is underneath a radial inward positioned magnet and hold	10 s	-	-	“
43	Move cartridge underneath radial inward magnet with -0.5°/s until magnet is at the centre of elution chamber	-	-	-	“
44	Transfer beads into elution chamber	0 s	-30 Hz	10 Hz/s	“
45	Pull beads radially inwards	16 s	10 Hz	5 Hz/s	“
46	Sediment beads	10 s	15 Hz	5 Hz/s	“
47	Repeat steps 45 to 46 19 times	-	-	-	“
48	Initiate bead transfer	0 s	5 Hz	5 Hz/s	“
49	Move cartridge until radial inward magnet is between elution chamber and detection chamber 2	15 s	-	-	“
50	Move cartridge underneath radial outward magnet with 0.5°/s until magnet is at the centre of washing chamber 2	-	-	-	“
51	Transfer beads into washing chamber 2	0 s	30 Hz	10 Hz/s	“
52	Heat up for temperature change rate actuated siphon priming	0 s	“	“	55 °C
53	Decelerate for fast air pressure release via elution chamber	30 s	5 Hz	5 Hz/s	“
54	Accelerate to balance liquid levels	10 s	15 Hz	5 Hz/s	“
55	Decelerate for temperature change rate actuated siphon priming	0 s	8 Hz	5 Hz/s	“
56	Cool down for temperature change rate actuated siphon priming	15 s	“	“	30 °C

57	Accelerate to transport elution into transport chamber	60 s	40 Hz	5 Hz/s	"
58	Accelerate to increase pneumatic pressure	10 s	60 Hz	5 Hz/s	"
59	Decelerate to transport liquid into mixing chamber	0 s	5 Hz	30 Hz/s	"
60	Heat up to transport residual liquid and first bubble mixing with pre-stored reagents	60 s	"	"	55 °C
61	Accelerate to avoid early siphon priming	0 s	50 Hz	5 Hz/s	"
62	Cool down to initiate second bubble mixing step	55 s	"	"	30 °C
63	Heat up for bubble mixing	0 s	"	"	55 °C
64	Decelerate to allow bubble mixing	30 s	5 Hz	5 Hz/s	"
65	Accelerate to balance liquid levels	10 s	20 Hz	5 Hz/s	"
66	Decelerate for temperature change rate actuated siphon priming	0 s	5 Hz	5 Hz/s	"
67	Cool down for temperature change rate actuated siphon priming	0 s	"	"	35 °C
68	Accelerate to generate monodisperse droplets inside detection chamber 1 and 2	180 s	40 Hz	5 Hz/s	"
69	Decelerate to transfer liquids onto steps by capillary forces inside of detection chamber 1 and 2	30 s	3 Hz	5 Hz/s	"
70	Heat up to start LAMP	3600 s	"	"	64 °C

Proviral load calculations

The number of peripheral blood-mononuclear cells (PBMC) counts $2x10^6$ cells/ml. This is equal to $2x10^3$ cells/µl or 10^5 cells/50 µl.

The percentage of HTLV-1 infected PBMC cells varies between HAM/TSP patients, HU patients and asymptomatic carriers [2]. The number of infected cells in a sample (50 µl blood) is calculated in the following:

- Mean value of HTLV-1 infected PBMC cells for asymptomatic carriers: 0.54 %
  In a 50 µl blood sample (10^5 cells): 540 infected cells
- Mean value of HTLV-1 infected PBMC cells for HAM/TSP patients: 11.63 %
  In a 50 µl blood sample (10^5 cells): 11630 infected cells
- Mean value of HTLV-1 infected PBMC cells for HU patients: 3.84 %
  In a 50 µl blood sample (10^5 cells): 3840 infected cells

Optical design of the POCT instrument

Figures S1 and S2 presented in this chapter have been published in an original research open access article under Attribution-NonCommercial 3.0 Unported (CC BY-NC 3.0) license: M. Schulz, S. Calabrese, F. Hausladen, H. Wurm, D. Drossart, K. Stock et al. (2020): Point-of-care testing system for digital single cell detection of MRSA directly from nasal swabs. In: Lab Chip 20 (14), S. 2549–2561.

An overview of the schematic depicition of the optical path of the optical unit installed in the POCT instrument is shown in Figure S1. The 3D-CAD inside view illustrating the components of the optical unit of the instrument is displayed in Figure S2. The POCT instrument is designed for the detection of three different fluorescence wavelengths which meet the requirements to detect the following dyes: FAM, TAMRA and Cy5. The specifications of the LEDs are given in Table S4. Further installed components: CCD camera model AV GE1650 from Allied Vision Technology GmbH (pixel size 7.4 µm, pixel number 1600*1200 pixel, quantum efficiency of the CCD sensor 55 % at 550 nm), telecentric lens model S5LPJ2999/M42 from Sill Optics GmbH &Co and a filter wheel model #88-171 from Edmund Optics Inc. equipped with excitation and emission filters from Semrock, IDEX Health & Science, LLC (Table S5). Detailed descriptions of the assembly and the operating principle are given in [3].

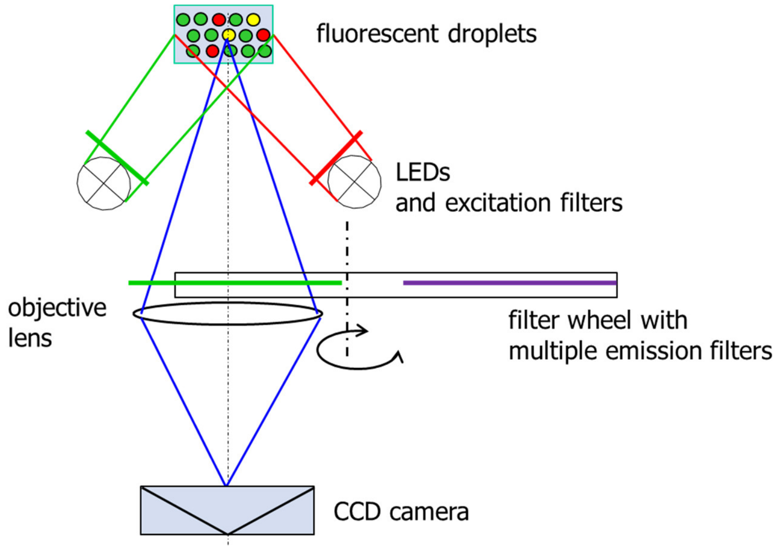

Figure S1. Schematic depiction of the optical path. Taken with permission from [3].

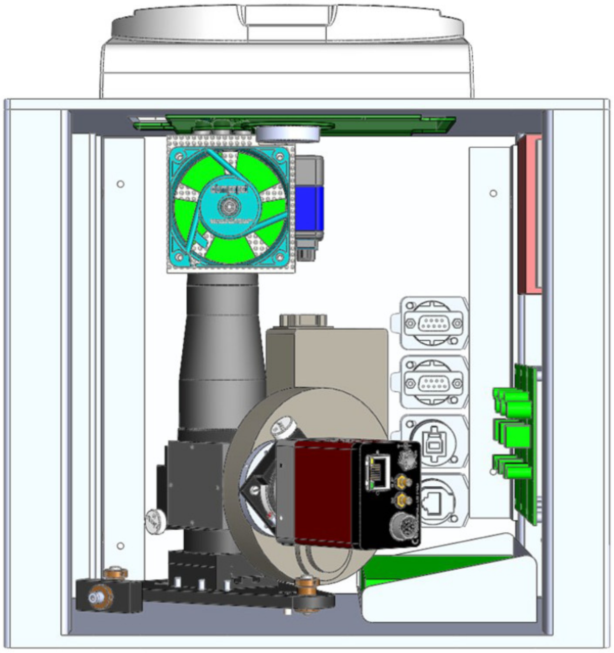

Figure S2. 3D-CAD inside view of the POCT instrument. Dimensions of the housing: 285 x 240 x 260 mm (w x d x h), weight: 15 kg. Taken with permission from [3].

Table S4. Specifications of the LEDs.

Model	Peak wave-length [nm]	Half bandwidth [nm]	Radiance angle (50 % I_v)	Radiant power [mW]	Emitter area [nm]
LB W5SM	465	25	120	264-580	1.0*1.0
LCG H9RN	520	-	120	470-872	0.98*0.98
LR W5SM	632	18	120	318-580	1.0*1.0

Table S5. Bandpass filters used to filter LED light (excitation filters), and bandpass filters for detection assembled in the filter wheel (emission filters). Within the transmission band, the transmission of the emission bandpass filters is > 93%.

Fluorescence light (dye)	Excitation filter	Emission filter
Green (FAM)	474/27 BrightLine HC	515/30 BrightLine HC
Orange (TAMRA)	554/23 BrightLine HC	595/31 BrightLine HC
Red (Cy5)	635/18 BrightLine HC	680/42 BrightLine HC

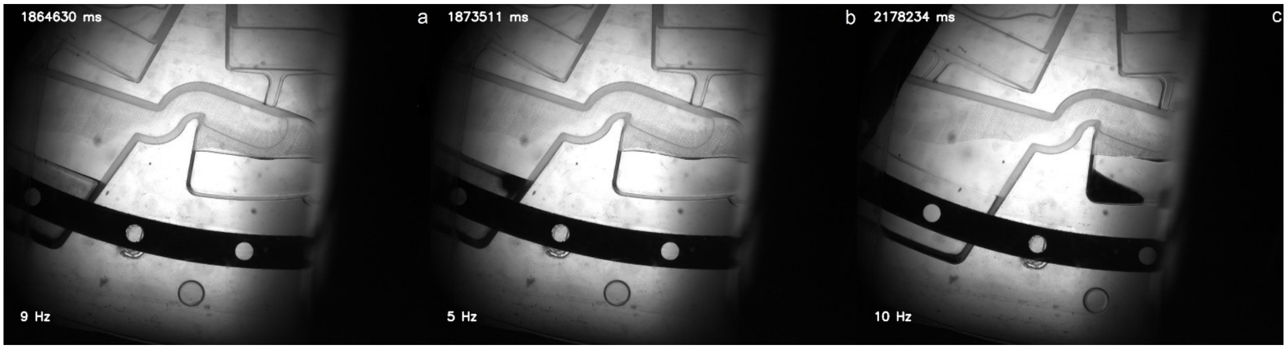

Figure S3. Stroboscopic images of the bead transfer. At the start, all beads were resuspended in the washing chamber 2 (a). When decreasing the rotational frequency, the magnetic forces from the stationary magnets started to be relevant compared to the centrifugal forces and the beads were attracted radially inwards (b). In the next step, the rotation of the cartridge was stopped and the cartridge was positioned underneath the static magnets. By slowly moving the cartridge in 0.5 °/s steps underneath the magnets, the beads were transferred from the washing chamber 2 into the elution chamber. The increasing rotational frequency then centrifuged the beads into the elution buffer (c).

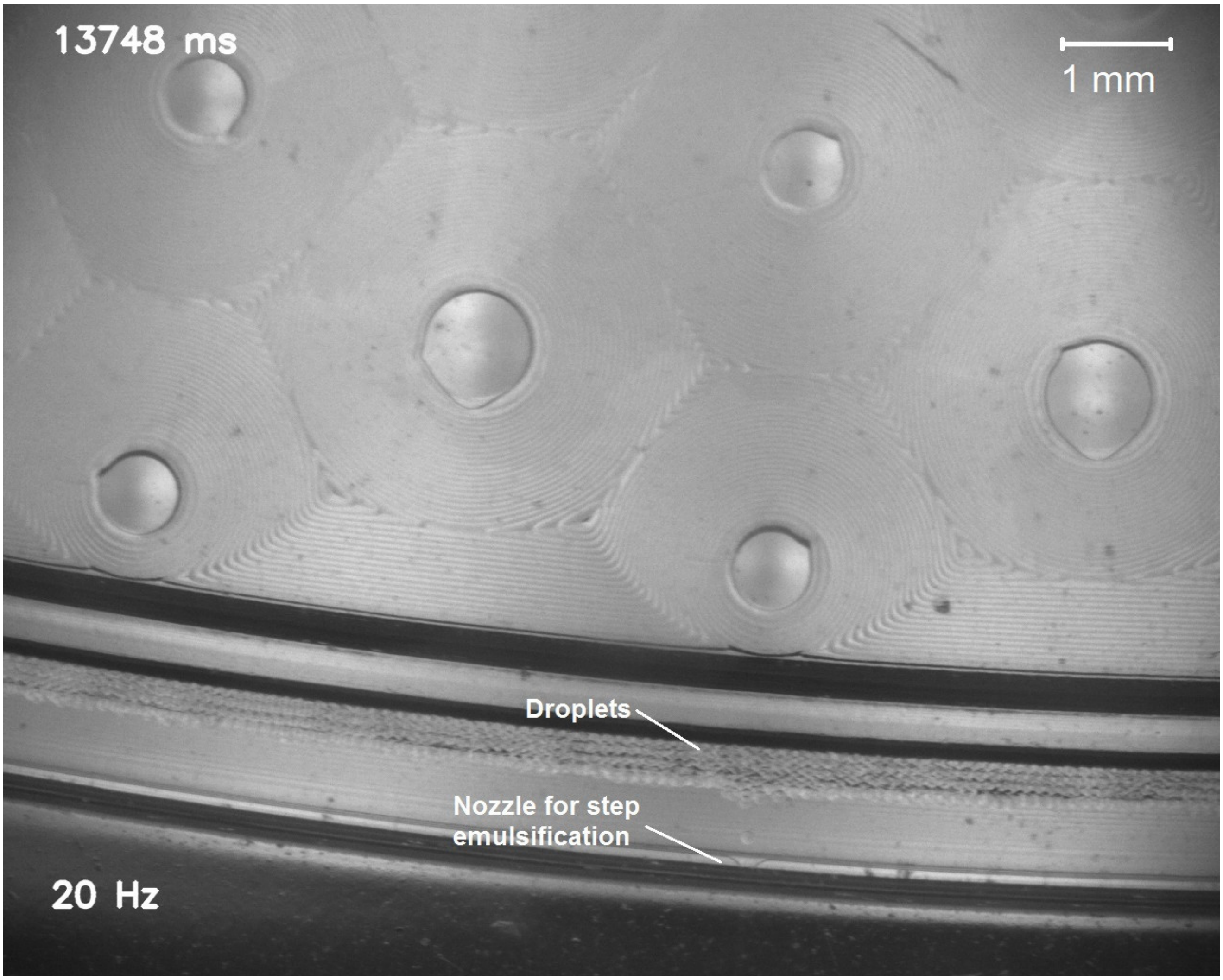

Figure S4. Stroboscopic image of the droplet generation. The LAMP mix was transferred into the detection chamber, which already contained the oil. At the nozzle, step emulsification occurred and droplets with a diameter of 100 μm were generated.

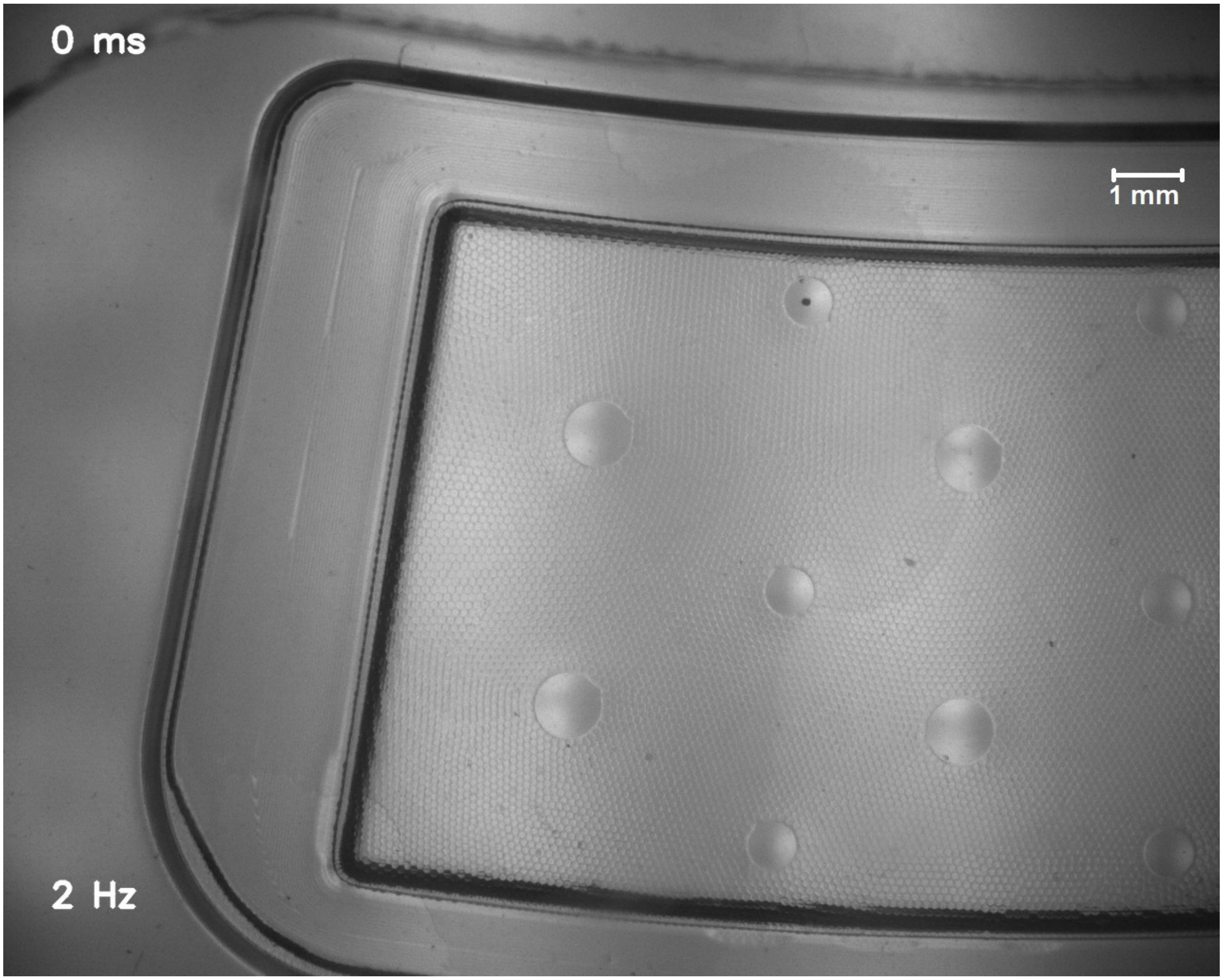

Figure S5. Stroboscopic image of the generated droplets. A monolayer formed on the shallow microfluidic structure.

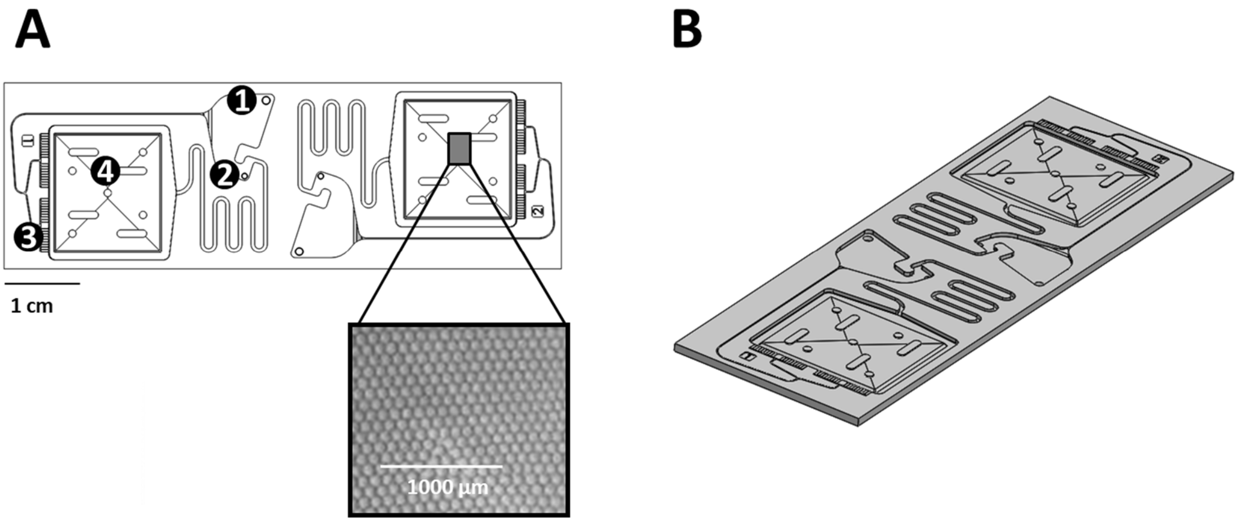

Figure S6. A) Layout of the microfluidic chip with two identical structures for droplet generation and digital LAMP: 1) sample inlet hole, 2) air vent hole, 3) nozzles for droplet generation by centrifugal step emulsification and 4) droplet collection and incubation chamber. The enlarged section displays an image of a monolayer of droplets with a diameter of 100 µm. B) 3D-CAD view of the microfluidic chip. The chip was manufactured by injection molding (cyclo-olefin polymers from Zeonor, Zeon Corp., Japan) at E.L.T. Kunststofftechnik & Werkzeugbau GmbH (Austria) and sealed with a pressure sensitive adhesive film (9795R, 3M Corp., United States). The droplet generation of the reaction mix was performed in a perfluorinated compound (5 % Pico-Surf 1 in Novec 7500, Dolomite Bio, United Kingdom) via rotation at 1500 rpm in a mini centrifuge. The digital RT-LAMP of HTLV-1 was performed in a blockcycler at 63 °C for 60 minutes and fluorescence readout took place in a microarray scanner. [4]

References

1. Becherer, L.; Bakheit, M.; Frischmann, S.; Stinco, S.; Borst, N.; Zengerle, R.; von Stetten, F. Simplified Real-Time Multiplex Detection of Loop-Mediated Isothermal Amplification Using Novel Mediator Displacement Probes with Universal Reporters. *Anal. Chem.* **2018**, *90*, 4741–4748, doi:10.1021/acs.analchem.7b05371.
2. Ono, A.; Mochizuki, M.; Yamaguchi, K.; Miyata, N.; Watanabe, T. Increased number of circulating HTLV-1 infected cells in peripheral blood mononuclear cells of HTLV-1 uveitis patients: a quantitative polymerase chain reaction study. *Br. J. Ophthalmol.* **1995**, *79*, 270–276, doi:10.1136/bjo.79.3.270.
3. Schulz, M.; Calabrese, S.; Hausladen, F.; Wurm, H.; Drossart, D.; Stock, K.; Sobieraj, A.M.; Eichenseher, F.; Loessner, M.J.; Schmelcher, M.; et al. Point-of-care testing system for digital single cell detection of MRSA directly from nasal swabs. *Lab Chip* **2020**, doi:10.1039/D0LC00294A.
4. Becherer, L.; Schulz, M.; Kuhn, H.; Bakheit, M.; Frischmann, S.; Zitz, F.; Borst, N.; Zengerle, R.; von Stetten, F. HIV-1 and HTLV-1 multiplex detection by digital mediator displacement LAMP. *Miniaturized Systems for Chemistry and Life Sciences (µTAS), Kaohsiung, Taiwan, November 11-15.* **2018**.
